# Calmodulin Interacts with the Sodium/Calcium Exchanger NCX1 to Regulate Activity

**DOI:** 10.1371/journal.pone.0138856

**Published:** 2015-09-30

**Authors:** Ai-Chuan Chou, Yu-Ten Ju, Chien-Yuan Pan

**Affiliations:** 1 Department of Life Science, National Taiwan University, Taipei, Taiwan; 2 Department of Animal Science and Technology, National Taiwan University, Taipei, Taiwan; 3 Graduate Institute of Brain and Mind Sciences, National Taiwan University, Taipei, Taiwan; Indiana University School of Medicine, UNITED STATES

## Abstract

Changes in intracellular Ca^2+^ concentrations ([Ca^2+^]_i_) are an important signal for various physiological activities. The Na^+^/Ca^2+^ exchangers (NCX) at the plasma membrane transport Ca^2+^ into or out of the cell according to the electrochemical gradients of Na^+^ and Ca^2+^ to modulate [Ca^2+^]_i_ homeostasis. Calmodulin (CaM) senses [Ca^2+^]_i_ changes and relays Ca^2+^ signals by binding to target proteins such as channels and transporters. However, it is not clear how calmodulin modulates NCX activity. Using CaM as a bait, we pulled down the intracellular loops subcloned from the NCX1 splice variants NCX1.1 and NCX1.3. This interaction requires both Ca^2+^ and a putative CaM-binding segment (CaMS). To determine whether CaM modulates NCX activity, we co-expressed NCX1 splice variants with CaM or CaM_1234_ (a Ca^2+^-binding deficient mutant) in HEK293T cells and measured the increase in [Ca^2+^]_i_ contributed by the influx of Ca^2+^ through NCX. Deleting the CaMS from NCX1.1 and NCX1.3 attenuated exchange activity and decreased membrane localization. Without the mutually exclusive exon, the exchange activity was decreased and could be partially rescued by CaM_1234_. Point-mutations at any of the 4 conserved a.a. residues in the CaMS had differential effects in NCX1.1 and NCX1.3. Mutating the first two conserved a.a. in NCX1.1 decreased exchange activity; mutating the 3^rd^ or 4^th^ conserved a.a. residues did not alter exchange activity, but CaM co-expression suppressed activity. Mutating the 2^nd^ and 3^rd^ conserved a.a. residues in NCX1.3 decreased exchange activity. Taken together, our results demonstrate that CaM senses changes in [Ca^2+^]_i_ and binds to the cytoplasmic loop of NCX1 to regulate exchange activity.

## Introduction

The change in the intracellular Ca^2+^ concentration ([Ca^2+^]_i_) is an important signal that controls versatile cellular processes, and there are several mechanisms that maintain Ca^2+^ homeostasis. At the plasma membrane, Ca^2+^-pumps and Na^+^ gradient-dependent Ca^2+^ transporters are the two main pathways for exporting Ca^2+^ out of cells when [Ca^2+^]_i_ is elevated. In addition, the direction of the Na^+^ gradient-dependent Ca^2+^ transporters can be reversed to transport Ca^2+^ into the cytosol according to the electrochemical gradients of coupled ions [1,2,3]. Two different families of solute carriers are responsible for Na^+^ gradient-dependent Ca^2+^ transport: *SLC8*, a Na^+^/Ca^2+^ exchanger (NCX) with a stoichiometry of 3 Na^+^ in exchange for 1 Ca^2+^, and *SLC24*, a Na^+^/Ca^2+^-K^+^ exchanger (NCKX) with a stoichiometry of 4 Na^+^ in exchange for 1 Ca^2+^ and 1 K^+^ [2,4,5,6].

**Fig 1:**
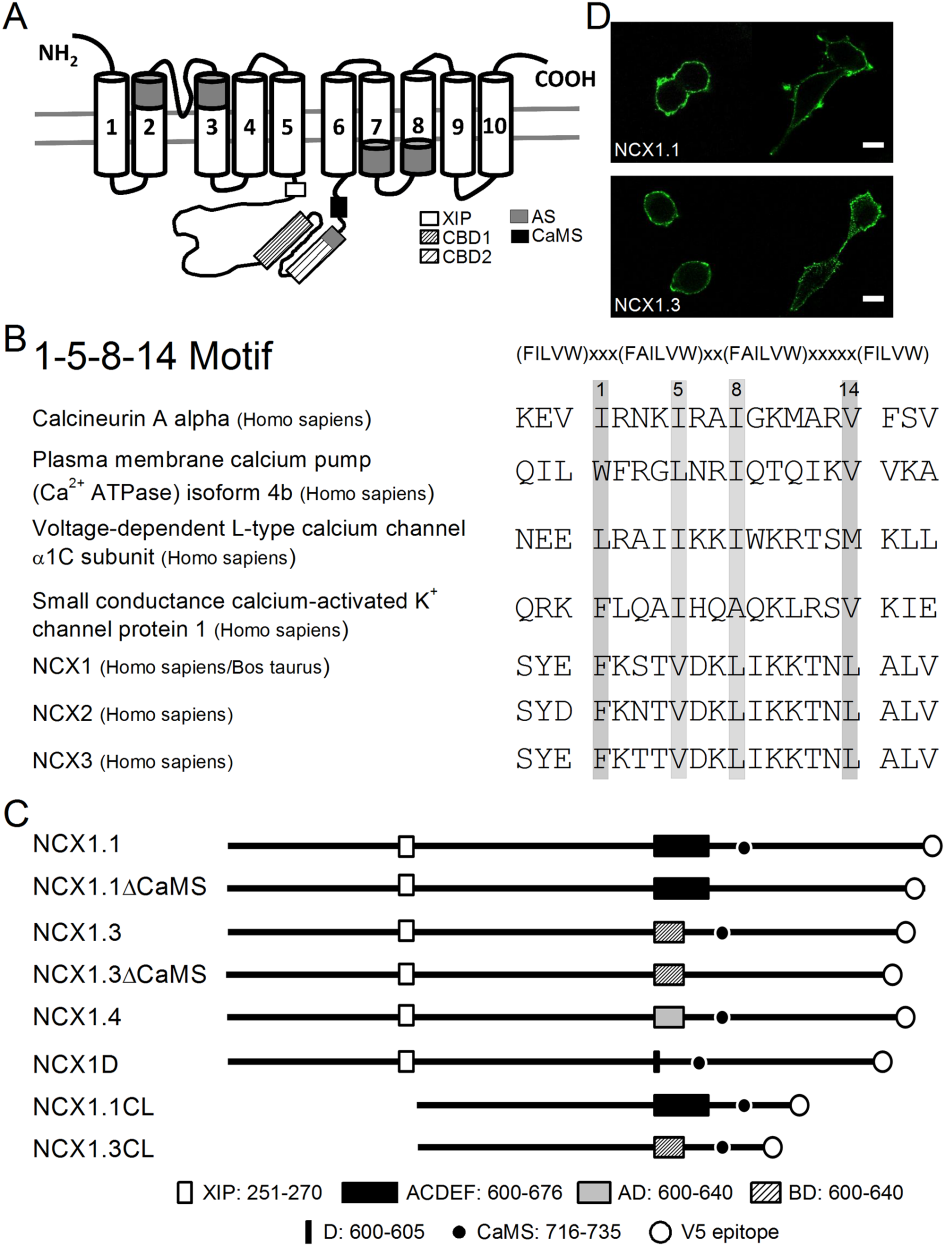
Schematic illustration of NCX1. A. Predicted secondary structure of NCX1. NCX has 10 transmembrane segments and the intracellular loop between the 5^th^ and 6^th^ transmembrane segments contains several modulatory regions including the exchanger inhibitory peptide (XIP), 2 α2-repeats (shaded regions), Ca^2+^ binding domains (CBD1 and CBD2), an alternative splicing site (AS), and the predicted CaM binding segment (CaMS). B. Sequence alignment of the CaM binding motif. The alignment includes the conserved 1-5-8-14 CaM binding sequences identified in human calcineurin (a.a. 405–424), human plasma membrane Ca^2+^-ATPase 4b (a.a. 1089–1108), human L-type Ca^2+^ channel (a.a. 1601–1620), and human small conductance Ca^2+^-activated K^+^ channel (a.a. 424–443), bovine/human NCX1.1 (a.a. 716–735), human NCX2 (a.a. 666–685), and human NCX3 (a.a. 663–681). Arrows indicates the conserved residues. C. The bovine NCX1 constructs. Empty box indicates the XIP (a.a. 216–270) and boxes filled with different patterns indicate the AS region with different exons. The filled circle indicates the putative CaMS and the empty circle at the C-terminus represents the V5 epitope tag. The number indicates the a.a residue in the NCX1.1. D. Immunostaining of NCX1.1 and NCX1.3 in non-permeabilized cells. HEK293T cells expressing NCX1.1 and NCX1.3 were stained with an antibody against the V5 epitope tagged at the C-terminus of these splice variants. Images were obtained using a Leica SP5 confocal microscope. Scale bar: 10 μm

NCX1 is composed of 970 a.a. residues and is predicted to have 10 transmembrane segments, with a large intracellular loop between the 5^th^ and 6^th^ transmembrane segments (TMs) (Fig 1A). The crystal structure of an archaebacterial NCX analogue (NCX_Mj, from *Methanococcus jannaschii*) was recently resolved, revealing 10 TMs [7,8]. A reexamination of the hydropathy profile of mammalian NCX1 indicates that this protein likely also has 10 TMs [9]. At the N-terminal of the intracellular loop, an XIP (exchanger inhibitory peptide) segment attenuates the Na^+^-dependent inactivation of NCX1 [10]. This is followed by a catenin-like domain whose function is not yet clear. In the C-half of the loop, there are two Ca^2+^ binding domains, CBD1 (K_d_ ~ 0.2 μM) and CBD2 (K_d_ ~ 5 μM), related to the activation of exchange activity [11,12,13]. The C-terminal of CBD2 contains an alternative splicing region that determines the stability of CBD2 [14].

Calmodulin (CaM) has 4 EF-hand Ca^2+^-binding motifs and binds to a variety of proteins to regulate different physiological activities and relay Ca^2+^ signals. CaM binds to the IQ motifs located at the C-termini of voltage-gated Na^+^ and Ca^2+^ channels to regulate channel activity in a Ca^2+^-dependent manner [15]. At the post-synaptic membrane, the influx of Ca^2+^ through NMDA receptors and Ca^2+^ channels activates the CaM-mediated modulation of long-term plasticity [16]. CaM binds to the 1-5-8-14 motif of the Ca^2+^-ATPase and enhances pump activity [17]. However, it is not clear whether CaM regulates the activity of NCX1.

In this study, we used glutathione S-transferase (GST)-CaM as a bait and, in the presence of Ca^2+^, pulled down the intracellular loop of NCX1 alternative splice variants containing the putative CaM binding segment (CaMS) from cell lysates. Deleting the CaMS or exon A/B in the alternative splicing region significantly reduces exchange activity. Mutations in conserved a.a. residues in the CaMS decreased exchange activity and rendered the exchanger sensitive to co-expression of CaM or the Ca^2+^-binding deficient CaM mutant, CaM_1234_. Our results demonstrate that CaM binds to NCX1 and regulates exchange activity.

## Materials and Methods

### Chemicals

Lipofectamine 2000, mouse monoclonal antibodies against the V5 epitope, CaM, and mouse IgG, Dulbecco’s Modified Eagle’s Medium, and other chemicals for cell culture were obtained from Invitrogen Inc. (Carlsbad, CA, USA). Fura-2 acetomethoxylmethyl ester (Fura-2 AM) was purchased from TefLabs (Austin, TX, USA). Unless otherwise indicated, all additional chemicals were purchased from Sigma-Aldrich Inc. (St. Louis, MO, USA).

### Plasmid preparation

NCX1.1 and NCX1.3 constructs were provided by Dr. Lung-Sen Kao (National Yang-Ming University, Taipei, Taiwan) [18]. For antibody recognition, we linked a V5 epitope to the C-terminus of each construct. To obtain NCX1 mutants, we used PfuUltra High-Fidelity DNA Polymerase AD (Agilent Technologies, USA) for site-directed mutagenesis. The paired primers used for the point-mutagenesis were: 5'-GAA TCC TAC GAG GCC AAG AGT ACC GTG GAC-3' (F1A); 5'-TTC AAG AGT ACC GCG GAC AAA CTG ATT AAG-3' (V5A); 5'-AGT ACC GTG GAC AAA GAC ATT AAG AAG ACA AAC-3' (L8D) and 5'-ATT AAG AAG ACA AAC GAC GCC CTC GTG GTT GGG-3' (L14D). The underlined bases in these primers indicate the mutated nucleotides. In the cytosolic loop construct, NCX1.1CL and NCX1.3CL, we added a V5 epitope to the C-terminus with the following mutagenic oligonucleotides: 5'-GAA GGT TTG CTT GAA TTC TTC TTC TTC CCC-3' (EcoRI), 5'-GCC GTT CCA GTA CTC TGT CGG GGG-3', 5'-ACA GAG TAC TGG AAC GGC ATA AAA GGC TTC GAT GCT-3', and 5'-GGG CCC TCT AGA CTA GCC GCC ACC-3' (XbaI).

NCX1 has 6 small exons (A, B, C, D, E, and F) involved in alternative splicing. To maintain an open reading frame, exons A and B are mutually exclusive; the other exons can be used in a variety of combinations (Fig 1C). The NCX1.1 splice variant has exons ACDEF; NCX1.3 has BD; and NCX1.4 contains AD. To characterize the importance of the mutually exclusive exons A and B, we constructed a clone containing only exon D (NCX1D). To characterize the importance of the CaMS, we deleted this region from NCX1.1 (NCX1.3ΔCaMS) and NCX1.3 (NCX1.3ΔCaMS). For pull-down assays, we cloned loop regions containing different exons but without the XIP (NCX1.1CL and NCX1.3CL). The V5 epitope at the C-terminus of these clones is for antibody recognition.

### Transfection of HEK293T cells

For transient expression of the exchanger protein in HEK293T cells grown in a 12-well plate, we mixed plasmids (1 μg total, including 0.1 μg of GFP plasmid) with Lipofectamine 2000 according to the manufacturer’s instructions. We used GFP fluorescence to identify transfected cells and performed experiments 24~36 hours after the transfection.

### Calcium imaging

To elevate the intracellular Na^+^ concentration, we incubated cells in Hank’s Balanced Salt Solution (HBSS, 130 mM NaCl, 2 mM KCl, 2.2 mM CaCl_2_, 1 mM MgCl_2_, 5.6 mM glucose, and 10 mM HEPES, pH 7.3) containing the Na^+^/K^+^-ATPase inhibitor, ouabain (100 μM), and fura-2 AM (5 μM) for 40 min at RT. We then mounted the cells on the stage of a Nikon Ti inverted microscope; to activate the reverse mode NCX exchange activity (rNCX), we locally perfused cells with NMG buffer (130 mM N-methyl-D-glucosamine (NMG), 2.2 mM CaCl_2_, 1 mM MgCl_2_, 5.6 mM glucose, and 10 mM HEPES, pH 7.3) to reverse the Na^+^ gradients. The buffer was puffed onto cells from a micropipette, with a tip opening of approximately 1 μm, positioned approximately 20 μm from a cell for 60 s at 3 psi, controlled by a Picospritzer III (Parker Instrument Inc., Parker Hannifin, Fairfield, NJ, USA). The excitation for fura-2 was provided by a DG4 (Sutter Inc., CA, U.S.A.) and emissions were collected by an EM-CCD camera (Photometrics, AZ, USA). The whole system was controlled by Nikon AIS Elements software. The fluorescence intensity ratios were converted into [Ca^2+^]_i_ by the previously reported protocol [19].

### Immunostaining

For antibody staining, we incubated cells in phosphate buffered saline (PBS, 137 mM NaCl, 2.7 mM KCl, 10 mM Na_2_HPO_4_, and 2 mM KH_2_PO_4_) containing 4% paraformaldehyde for 20 min at RT. After a PBS wash, we placed the cells in PBS containing 0.5% Triton X-100 and 1% BSA for 1 hr. We then washed the cells with PBS and incubated them in PBS containing a primary mouse antibody against the V5 epitope (1:500 dilution) at 4°C overnight. After another PBS wash, we incubated the cells in PBS containing the secondary antibody (goat anti-mouse IGg) conjugated to Alexa Fluor 488 at a 1:2000 dilution for 1 hr at RT. To label F-actin and DNA, we placed the cells in PBS containing rhodamine-conjugated phalloidin (10 μM) and 4', 6-diamidino-2-phenylindole (DAPI) (0.5 μg/mL) for 20 min at RT. After a PBS wash, we visualized the fluorescence by imaging with a Leica SP5 confocal microscope. To keep the cell membrane intact, we did not fix the cells with paraformaldehyde until after binding the primary and secondary antibodies at 4°C. The incubation for primary antibody staining was 1 hr.

To visualize the plasma membrane, we coexpressed glycosylphosphatidylinositol (GPI)-tagged green fluorescence protein (GFP-GPI) (a gift from Dr. You-Tzung Chen, National Taiwan University) with NCX1 alternative splice variants. To analyze the colocalization of GFP-GPI with NCX1 splice variants, we used a plug-in in ImageJ software to calculate the product of the differences from the mean (PDM) [20]. For each pixel, PDM = (red intensity—mean red intensity) × (green intensity—mean green intensity) and we displayed positive image only. The more positive value indicates higher possibility in overlapping at that pixel.

### Protein extraction

We resuspended transfected HEK293T cells in PBS and lysed the cells by sonication. We centrifuged the lysates at 1,000 × *g* for 30 min and collected the supernatant for another round of centrifugation at 100,000 × *g* for 2 hr. We collected the supernatant as the cytosolic fraction and the pellet as the membrane fraction.

### GST pull-down assay

We purified the GST-fused CaM or CaM_1234_ according to the previously published protocol [21] and used a Bradford-based protein assay kit (Bio-Rad, CA, USA) to estimate concentration. To pull down interacting proteins, we incubated GST-fused proteins or GST (1 mg) with GSH-Sepharose 4B beads (GE Healthcare, U.S.A.) following the protocol suggested by the manufacturer. We mixed the beads with cell lysates at 4°C overnight in a Binding Buffer (100 mM NaCl, 50 mM MgCl_2_, 10% Glycerol, 0.1% Nonidet P-40, 0.2% BSA, 25 mM HEPES, and 1 mM DTT) containing a Protease Inhibitor Cocktail (Set V, 1:100 dilution, CalBiochem, La Jolla, California, USA). The proteins bound to the beads were analyzed by SDS-PAGE followed by Western blot.

### Data analysis

Data are presented as the Mean ± SEM from at least three different batches of cells and analyzed by one-way ANOVA with Fisher's post hoc test. Differences were considered significant when the *p* value was under 0.05. The number of cells (n) indicates the total number of cells tested.

## Results

### Putative CaM binding segment in the NCX1 cytoplasmic loop

The predicted NCX structure has a large cytoplasmic loop, located between the 5^th^ and 6^th^ TMs, that contains several regulatory motifs (Fig 1A). Recent structural studies imply that there are 5, instead of 4, TMs after the large intracellular loop [7,8]. Aligning bovine NCX1 with the conserved CaM-binding 1-5-8-14 motif identified in calcineurin [22], the plasma membrane Ca^2+^-ATPase [17], L-type Ca^2+^ channel [23], and the small conductance Ca^2+^-activated K^+^ channel [24] shows a conserved 1-5-8-14 segment in NCX1 (CaMS, a.a. residues 716–735) (Fig 1B) positioned after the alternative splicing region. The other two NCX isoforms, NCX2 and NCX3, also contain this putative 1-5-8-14 motif at the C-terminus of the large cytosolic loop; however, no predicted CaM binding motif is present in NCKX isoforms (Calmodulation database and meta-analysis target site predictor, http://cam.umassmed.edu/).

To verify the orientation of the NCX1 C-terminus, we stained non-permeabilized HEK293T cells expressing NCX1.1 or NCX1.3 with an antibody against the V5 epitope, thereby preventing the antibody from diffusing into the cytosol (Fig 1D). Images show that the fluorescence signal localizes to the perimeter of the transfected cells when the cell membrane is intact. In contrast, cells expressing only the intracellular loop tagged with V5 did not show fluorescence staining in non-permeabilized cells, whereas fluorescence was visible after cells were permeabilized (S1 Fig). These results indicate that the C-terminus of NCX1 is extracellular.

### CaM interacts with the NCX1 cytoplasmic loop

**Fig 2:**
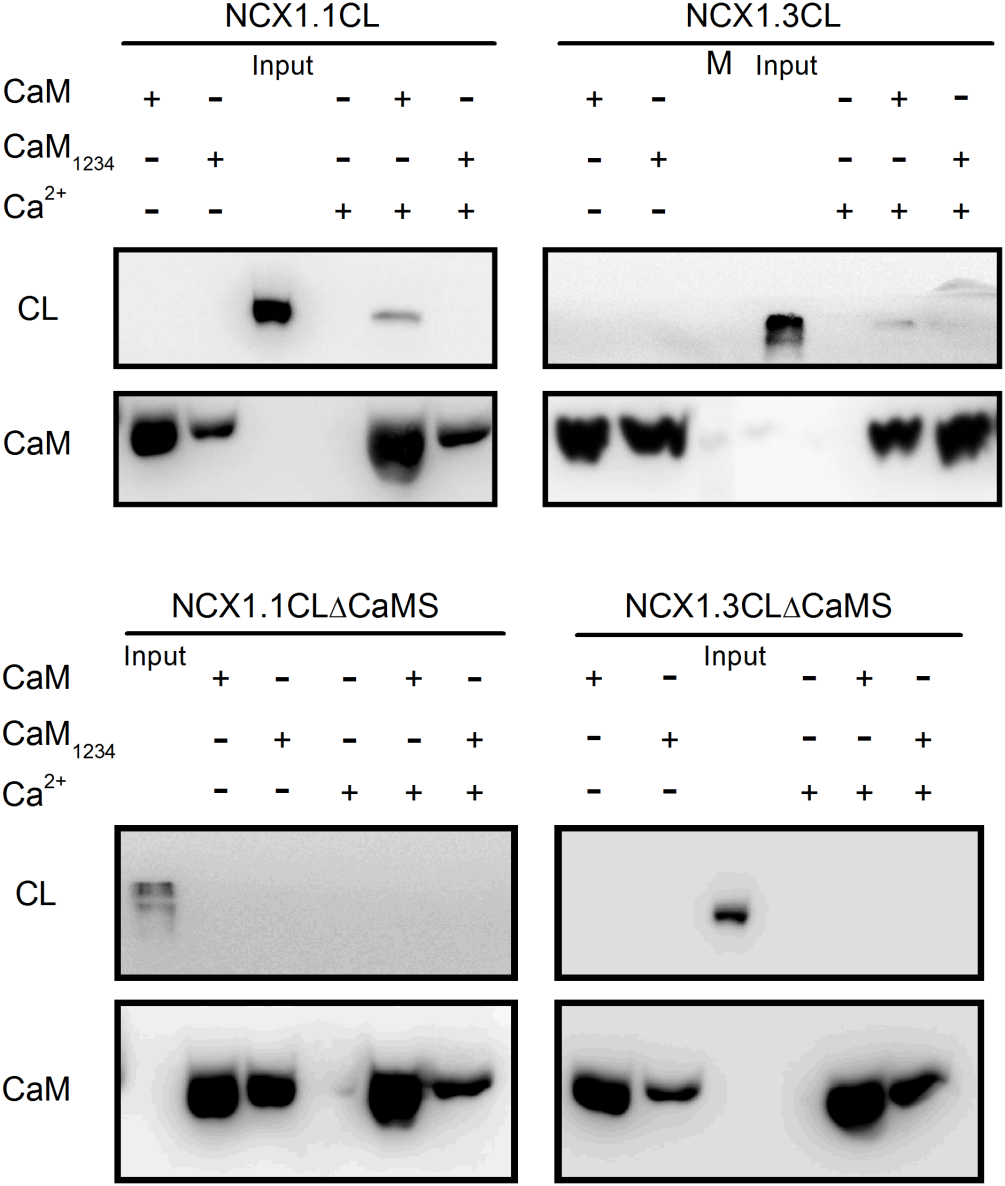
CaM interacts with the NCX1 cytosolic loop. Purified GST-CaM or GST-CaM_1234_ was used as bait to pull down intracellular loops subcloned from NCX1.1 or NCX1.3 with (A. NCX1.1CL: a.a. 288–805 and B. NCX1.3CL: a.a. 288–769) or without the CaMS (C. NCX1.1CLΔCaMS and D. NCX1.3CLΔCaMS). During some of the binding reaction, Ca^2+^ (2 mM) was included in the buffer and it was omitted in other reactions. The interacting proteins were verified by Western blots with antibodies against the GST or V5 epitope. M indicates the m.w marker lane.

To determine whether NCX1 interacts with CaM, we used GST-CaM as the bait to pull down the cytoplasmic loops of NCX1.1 (NCX1.1CL: a.a. 288–805) and NCX1.3 (NCX1.3CL: a.a. 288–769) expressed in HEK293T cells (Figs 1C and 2). Western blots show that the antibody against the V5 epitope stains a protein with a molecular weight similar to the expected size (~70 kD) of NCX1.1CL or NCX1.3CL in the presence of Ca^2+^. In the absence of Ca^2+^ or CaM, no stained band was visualized. Neither GST-CaM nor GST-CaM_1234_ interacted with the cytoplasmic loop without the CaMS (NCX1.1CLΔCaMS and NCX1.3CLΔCaMS). These results suggest that CaM interacts specifically with the NCX1 cytoplasmic loop via the CaMS in a Ca^2+^-dependent manner.

### CaMS deletion affects membrane localization

**Fig 3:**
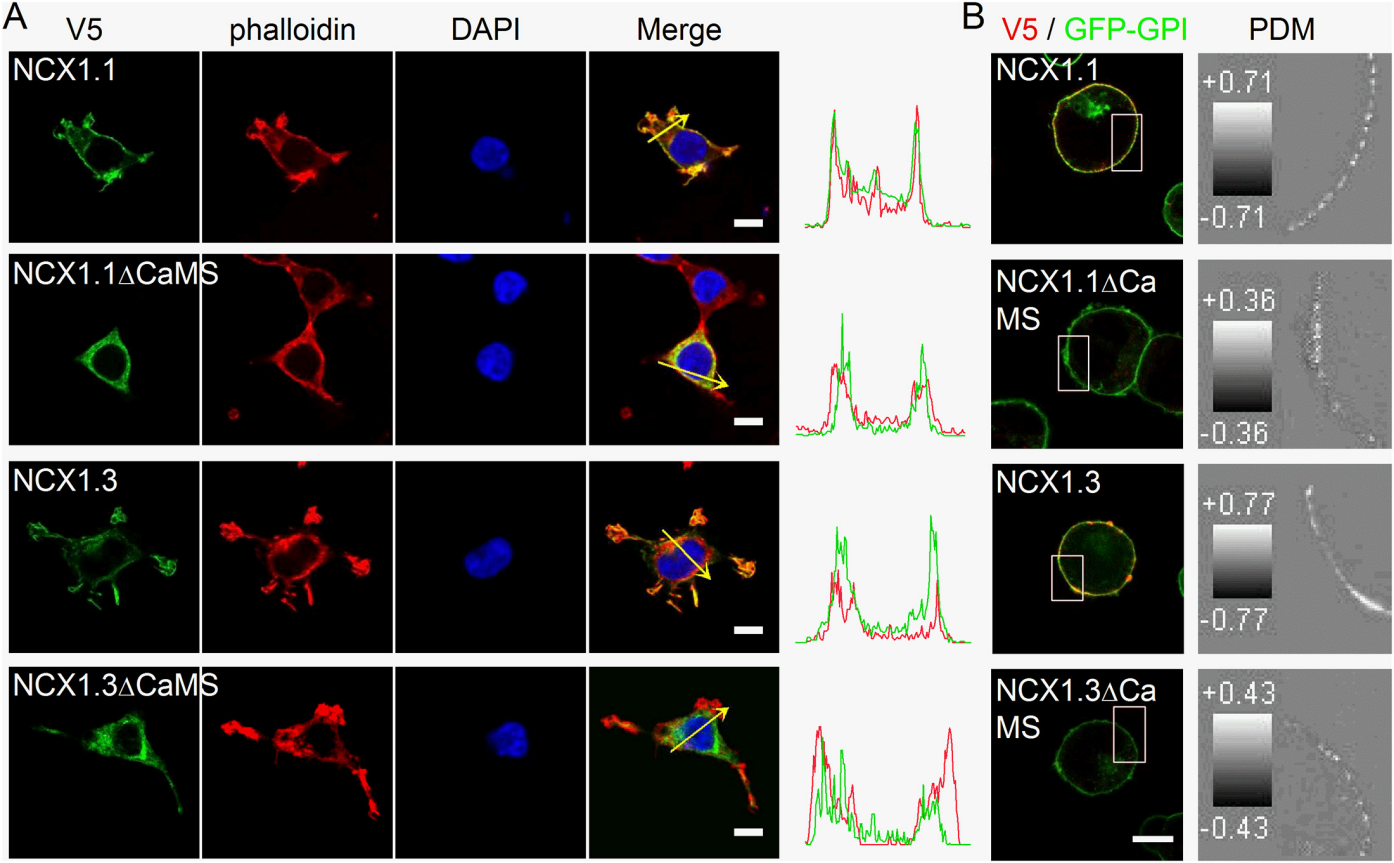
The CaMS affects the membrane localization of NCX1 splice variants. A. HEK293T cells expressing NCX1.1 or NCX1.3, with or without the CaMS (ΔCaMS), were permeabilized and stained with an antibody against the V5 epitope (Green) to label the exchanger. Phalloidin (Red) and DAPI (Blue) were used to visualize actin filaments and nuclear DNA, respectively. Images were obtained on a Leica SP5 confocal microscope. The intensity profiles plotted on the right indicate the red and green fluorescence intensity along the arrows in the corresponding merged images. B. Immunostaining of non-permeabilized HEK293T cells. Left, cells co-expressing NCX1 splice variants (Red) and GFP-GPI (Green). Right, PDM image of the boxed region shown to the left. The grey scale in each image represents the PDM value in each pixel. Scale bar: 10 μm

To characterize whether CaMS affects the localization of NCX1 splice variants, we expressed NCX1.1 and NCX1.3 with or without CaMS in HEK293T cells and stained the cells with an antibody against the V5 epitope (Fig 3). After fixation and permeabilization, the confocal images show that wild-type NCX1.1 and NCX1.3 are largely present at the plasma membrane compared with phalloidin staining, which illustrated the distribution of F-actin to the subplasmalemmal region. The line intensity profiles indicated that both NCX1.1 and NCX1.3 had an overlapping distribution with F-actin at the cell boundary. NCX1 mutants that lack the CaMS (NCX1.1ΔCaMS and NCX1.3ΔCaMS) localized to both the plasma membrane and the cytosolic region. Compared with the F-actin distribution shown by the line intensity profiles, both mutants without the CaMS localized mostly to the cytosolic side of the plasma membrane. To further confirm the membrane localization, we co-expressed the NCX1 splice variants and mutants with GFP-GPI, which encodes a GPI-anchored form of GFP that localized to the extracellular face of the plasma membrane, in HEK293T cells. We then stained the V5 epitope in non-permeabilized cells (Fig 3B). Both NCX1.1 and NCX1.3 were present at the membrane surface and overlapped with GFP-GPI. Under the same exposure settings, both mutants without CaMS displayed lower membrane surface expression levels than those of the corresponding wild-type proteins. The PDM images showed that the distributions of both NCX1.1 and NCX1.3 correlated well with GFP-GPI at the plasma membrane; in contrast, a lower correlation was observed in cells expressing NCX1.1ΔCaMS or NCX1.3ΔCaMS. In permeabilized cells, the fluorescence signals of the stained V5 and GFP-GPI were concentrated at the plasma membrane for NCX1.1 and NCX1.3; in contrast, the fluorescence signals in permeabilized cells expressing NCX1.1ΔCaMS or NCX1.3ΔCaMS were mostly distributed in a cytosolic region near the membrane (S2 Fig). Therefore, both F-actin and GFP-GPI staining revealed that NCX1 splice variants without the CaMS appeared to be localize to the cytosolic side of the membrane and partly on the cell membrane. These results suggest that CaMS plays an important role in the targeting of NCX1 splice variants to the plasma membrane.

### CaMS deletion decreases exchange activity

**Fig 4:**
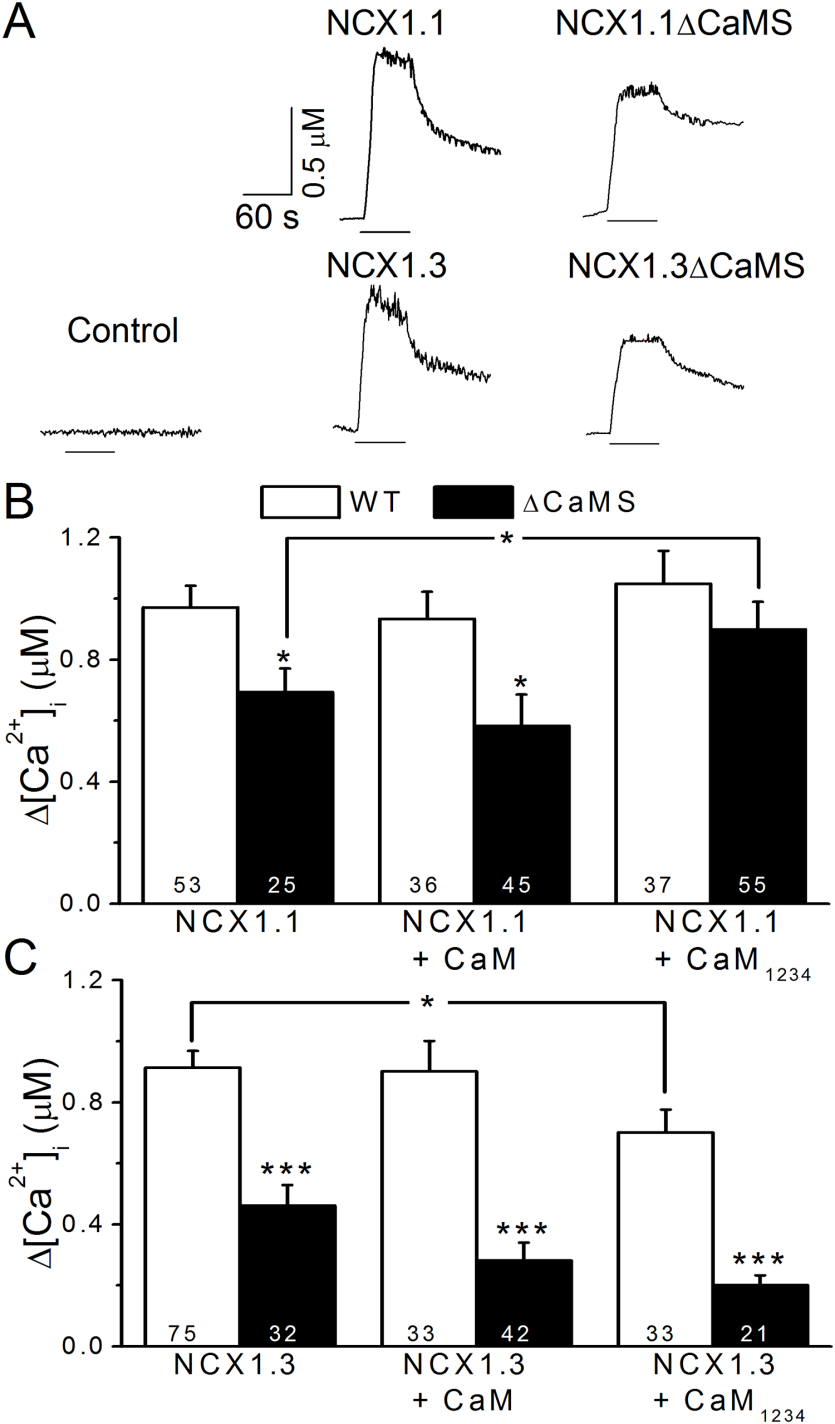
Deletion of the CaMS reduces exchange activity. HEK293T cells expressing NCX1 and mutants were treated with ouabain to elevate the intracellular Na^+^ concentration. Cells were then locally perfused with NMG buffer for 1 min to induce reverse-mode exchange activity. The [Ca^2+^]_i_ was calibrated based on changes in fura-2 fluorescence intensities. A. Representative [Ca^2+^]_i_ traces in single cells expressing different constructs. The black lines under each trace indicate the period of NMG perfusion. Cells transfected with a plasmid missing the exchanger were used as a control. B. The average elevation of [Ca^2+^]_i_ in cells expressing wild-type NCX1.1 (WT, empty columns) and NCX1.1ΔCaMS (ΔCaMS, filled columns) with CaM or CaM_1234_ co-expression. C. Average [Ca^2+^]_i_ responses in cells expressing NCX1.3 WT (empty columns) and NCX1.1ΔCaMS (filled columns) with CaM or CaM_1234_ co-expression. The digits in each column indicate the sample number. Data are the mean ± SEM pooled from three different sets of cells and analyzed by a one-way ANOVA with Fisher's post hoc test (*: *p* < 0.05, ***: *p* < 0.001 compared with WT).

To determine the importance of the CaMS on NCX1 activity, we expressed the splice variants in HEK293T cells and monitored [Ca^2+^]_i_ elevations induced by rNCX activity. To reverse the Na^+^ gradient, we treated the cells with ouabain to inhibit the Na^+^-pump and locally perfused a single cell with NMG buffer, in which Na^+^ was substituted with NMG. Fig 4A shows that during NMG perfusion, [Ca^2+^]_i_ rose quickly and then remained steady in cells expressing either NCX1.1 or NCX1.3; after the perfusion period, [Ca^2+^]_i_ gradually returned to basal levels. Despite receiving the same treatment, cells expressing only the vector did not show an elevation in [Ca^2+^]_i_. Cells expressing NCX1.1ΔCaMS or NCX1.3ΔCaMS showed a similar [Ca^2+^]_i_ response pattern, but the elevation was smaller compared with cells expressing the wild type. The average [Ca^2+^]_i_ change in cells expressing NCX1.1 and NCX1.3 was 971 ± 71 (n = 53) and 913 ± 55 (n = 75) nM, respectively. Without the CaMS, the [Ca^2+^]_i_ increases were significantly reduced to 694 ± 77 (n = 25, *p* < 0.05) and 462 ± 67 (n = 32 *p* < 0.001) nM in cells expressing NCX1.1ΔCaMS and NCX1.3ΔCaMS, respectively (Fig 4B and 4C).

To characterize the importance of CaM in regulating exchange activity, we co-expressed the NCX1 splice variants with wild-type CaM or the Ca^2+^-binding deficient mutant, CaM_1234_, in HEK293T cells and monitored the rNCX activity. The results show that, on average, neither CaM nor CaM_1234_ had a significant effect on NCX1.1 activity, with [Ca^2+^]_i_ increasing to 934 ± 88 (n = 36) and 1049 ± 108 (n = 37) nM, respectively. However, CaM_1234_, but not CaM, significantly enhanced the exchange activity of NCX1.1ΔCaMS to 899 ± 90 (n = 55, *p* < 0.05) nM.

For NCX1.3, CaM overexpression did not change the [Ca^2+^]_i_ response (902 ± 100 nM, n = 33), but CaM_1234_ significantly attenuated the response to 701 ± 75 (n = 33, *p* < 0.05) nM. NCX1.3ΔCaMS had a lower exchange activity than wild type (462 ± 67 nM, n = 32, *p* < 0.001). However, overexpression of either CaM or CaM_1234_ induced a significant decrease in exchange activity to 281 ± 59 (n = 42, *p* < 0.05) or 201 ± 32 nM (n = 21, *p* < 0.01), respectively. These results indicate that the CaMS motif is required for both NCX1.1 and NCX1.3 to maintain exchange activity and that CaM has differential effects on NCX1.1 and NCX1.3.

### Exons A and B are involved in CaM-mediated regulation

**Fig 5:**
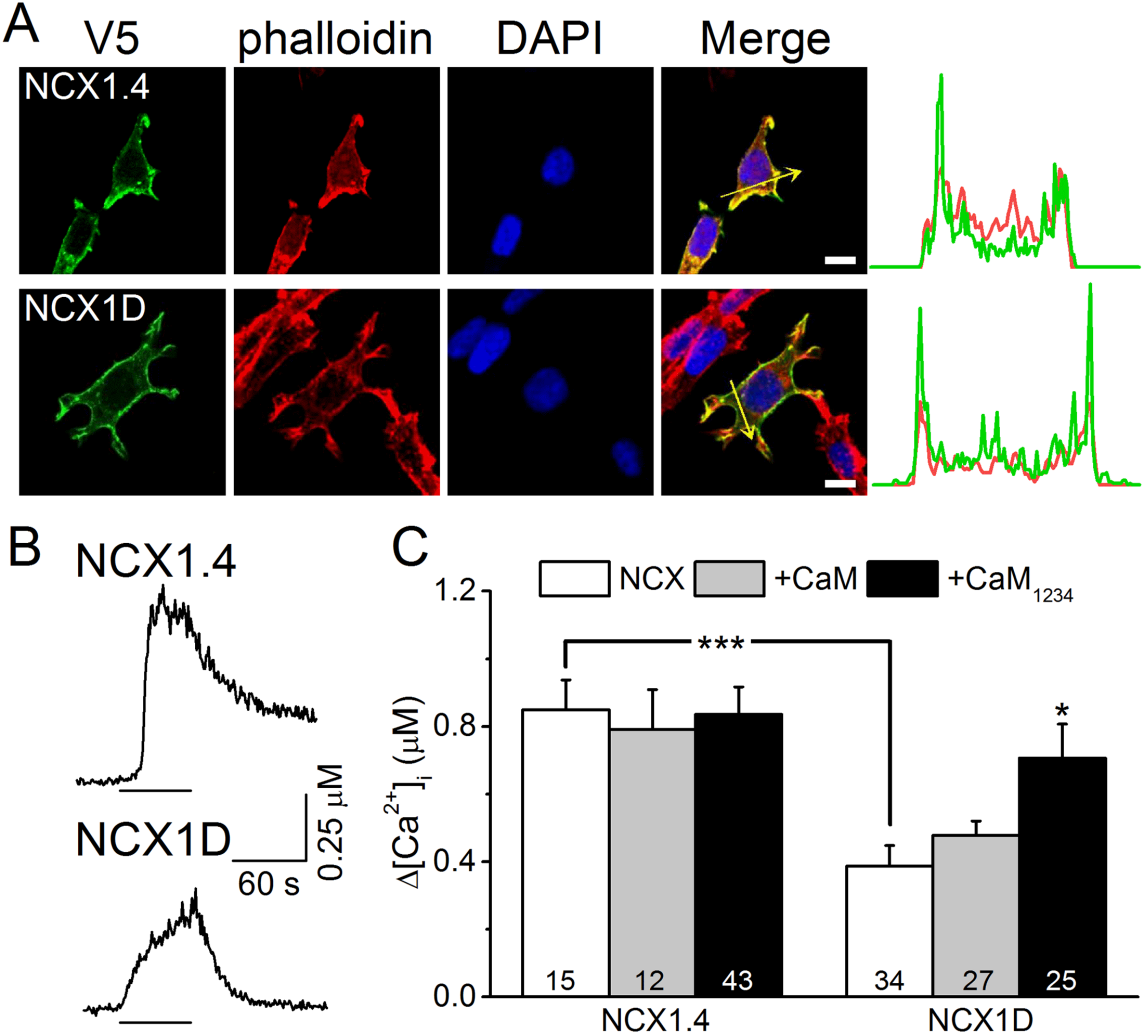
Deletion of exon A/B attenuates exchange activity. To activate the rNCX activity in HEK293T cells expressing NCX1.4 or NCX1D, we treated them with ouabain and then perfused with NMG buffer. The [Ca^2+^]_i_ was calibrated based on the changes in fura-2 fluorescence intensities. A. Localization of NCX1.4 and NCX1D. Cells expressing NCX1.4 (upper row) or NCX1D (lower row) were stained with V5 antibody (Green), phalloidin (Red), and DAPI (Blue) to visualize the exchanger, actin filaments, and nuclear DNA, respectively. The intensity profiles plotted on the right indicate intensity of the red and green fluorescence signals along the arrows indicated in the corresponding merged images. Scale bar: 10 μm. B. Representative [Ca^2+^]_i_ response traces from cells expressing NCX1.4 or NCX1D. The lines under each trace indicate the period of NMG perfusion. C. Average [Ca^2+^]_i_ changes in cells expressing NCX1.4 and NCX1D with co-expression of CaM or CaM_1234_. Data presented are the mean ± SEM pooled from three different batches of cells and analyzed by a one-way ANOVA with Fisher's post hoc test (*: *p* < 0.05, ***: *p* < 0.001 compared with the group without co-expression).

The difference between NCX1.1 and 1.3 are the mutually exclusive exons A and B, which are important for the Ca^2+^-chelating ability of the CBD2 motif [11,14]. To characterize the role of these exons in CaM-related regulatory effects, we constructed two NCX1 splice variants containing exons A and D (NCX1.4) or only exon D (NCX1D) for use in rNCX activity assays (Fig 5). Immunostaining shows that both NCX1.4 and NCX1D localize to the plasma membrane compared with the distribution of F-actin. Line intensity profiles confirm that the distributions of NCX1.4 and NCX1D were concentrated at the cell membrane. During NMG perfusion, cells expressing NCX1.4 and NCX1D showed elevated [Ca^2+^]_i_; however, NCX1D had a smaller [Ca^2+^]_i_ elevation than that of NCX1.4. The average [Ca^2+^]_i_ change in cells expressing NCX1.4 was 850 ± 87 nM (n = 15); CaM and CaM_1234_ co-expression did not affect [Ca^2+^]_i_, with [Ca^2+^]_i_ reaching 790 ± 119 (n = 12) and 836 ± 81 (n = 43) nM, respectively. In cells expressing NCX1D, the average [Ca^2+^]_i_ elevation was 387 ± 61 nM (n = 34, *p* < 0.001), which was significantly less than that in cells expressing NCX1.4. CaM co-expression did not affect the exchange activity of NCX1D (478 ± 43 nM, n = 27), but CaM_1234_ overexpression enhanced the [Ca^2+^]_i_ changes to 707 ± 101 nM (n = 25, *p* < 0.05). These results suggest that exons A and B support exchange activity and are involved in the CaM-mediated modulation of NCX1 activity.

### Mutating the conserved a.a. in the CaMS of NCX1.1 affects exchange activity

**Fig 6:**
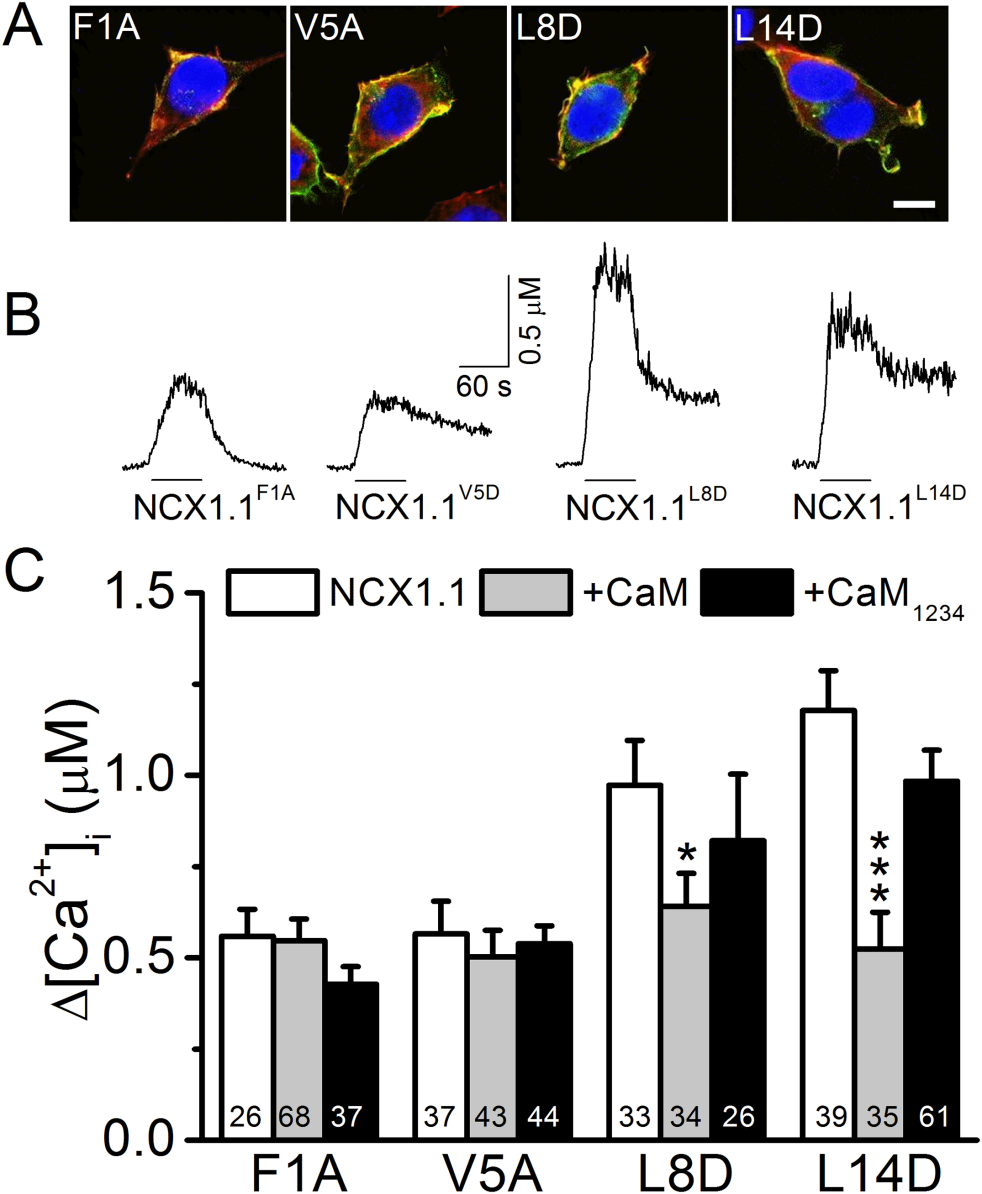
Each of the conserved a.a. residues in the NCX1.1 CaMS has differential effects on exchange activity. The conserved 1^st^, 5^th^, 8^th^, and 14^th^ a.a. residues of the CaMS in NCX1.1 was independently mutated from F, V, L, and L, to A (F1A), A (V5A), D (L8D), and D (L14D), respectively. We measured the reverse-mode exchange activity of HEK293T cells expressing these constructs with or without co-expression of CaM or CaM_1234_. A. Merged images of cells expressing various NCX1.1 mutants stained with phalloidin (Red), DAPI (Blue), and an antibody against the V5 epitope tag (Green) to visualize actin filaments, nuclear DNA, and the exchanger, respectively. Scale bar: 10 μm. B. Representative [Ca^2+^]_i_ responses from single HEK293T cells expressing various constructs. The line under each trace indicates the period of NMG perfusion. C. Average [Ca^2+^]_i_ elevations in cells expressing the various NCX1.1 mutants with or without co-expression of CaM or CaM_1234_. The digits in each column indicate the number of cells in each group. Data are the mean ± SEM pooled from three different batches of cells and analyzed by a one-way ANOVA with Fisher's post hoc test (*: *p* < 0.05, ***: *p* < 0.001 when compared with the corresponding group expressing only the NCX1.1 mutant).

To further characterize the importance of the CaMS in CaM-mediated regulation, we mutated the 4 conserved a.a. residues (Fig 1B) in the CaMS from F, V, K, and L to A, A, D, and D (named F1A, V5A, L8D, and L14D) [25] and monitored rNCX activity (Fig 6). Immunostaining for the V5 epitope at the C-termini of these mutants showed a concentrated distribution at the cell boundary, overlapping with F-actin. The line intensity profiles implied that these mutants were located at the cell membrane (S4 Fig). These mutants also showed rNCX activity; however, NCX1.1^F1A^ and NCX1.1^V5A^ had lower [Ca^2+^]_i_ elevation levels compared with the other two mutants. The averages show that mutants NCX1.1^F1A^ and NCX1.1^V5A^ had a significantly reduced change in [Ca^2+^]_i_ to 559 ± 74 (n = 26, *p* < 0.01) and 566 ± 90 (n = 37, *p* < 0.01) nM, respectively, compared with NCX1.1 (971 ± 71 nM, Fig 4B). CaM and CaM_1234_ co-expression did not affect [Ca^2+^]_i_ changes in cells expressing NCX1.1^F1A^, with [Ca^2+^]_i_ levels of 547 ± 59 (n = 68) and 428 ± 48 (n = 37) nM, respectively, or NCX1.1^V5A^, with [Ca^2+^]_i_ levels of 503 ± 73 (n = 43) and 539 ± 49 nM (n = 44), respectively.

The [Ca^2+^]_i_ changes in cells expressing NCX1.1^L8D^ and NCX1.1^L14D^ were 973 ± 123 (n = 33) and 1178 ± 108 (n = 39) nM, respectively, which are comparable to the changes in cells expressing NCX1.1. CaM co-expression significantly reduced the [Ca^2+^]_i_ changes in cells expressing NCX1.1^L8D^ and NCX1.1^L14D^ to 641 ± 90 (n = 34, *p* < 0.05) and 523 ± 101 (n = 35, *p* < 0.001) nM, respectively. CaM_1234_ co-expression did not affect the [Ca^2+^]_i_ changes in cells expressing NCX1.1^L8D^ and NCX1.1^L14D^ (820 ± 182 (n = 26) and 984 ± 85 (n = 61) nM). These data reveal that the first two conserved a.a. in NCX1.1 are important to support exchange activity; the 3^rd^ and 4^th^ a.a. mutations make the exchanger sensitive to CaM overexpression.

### Mutating the middle 2 conserved a.a. in the CaMS of NCX1.3 reduces exchange activity

**Fig 7:**
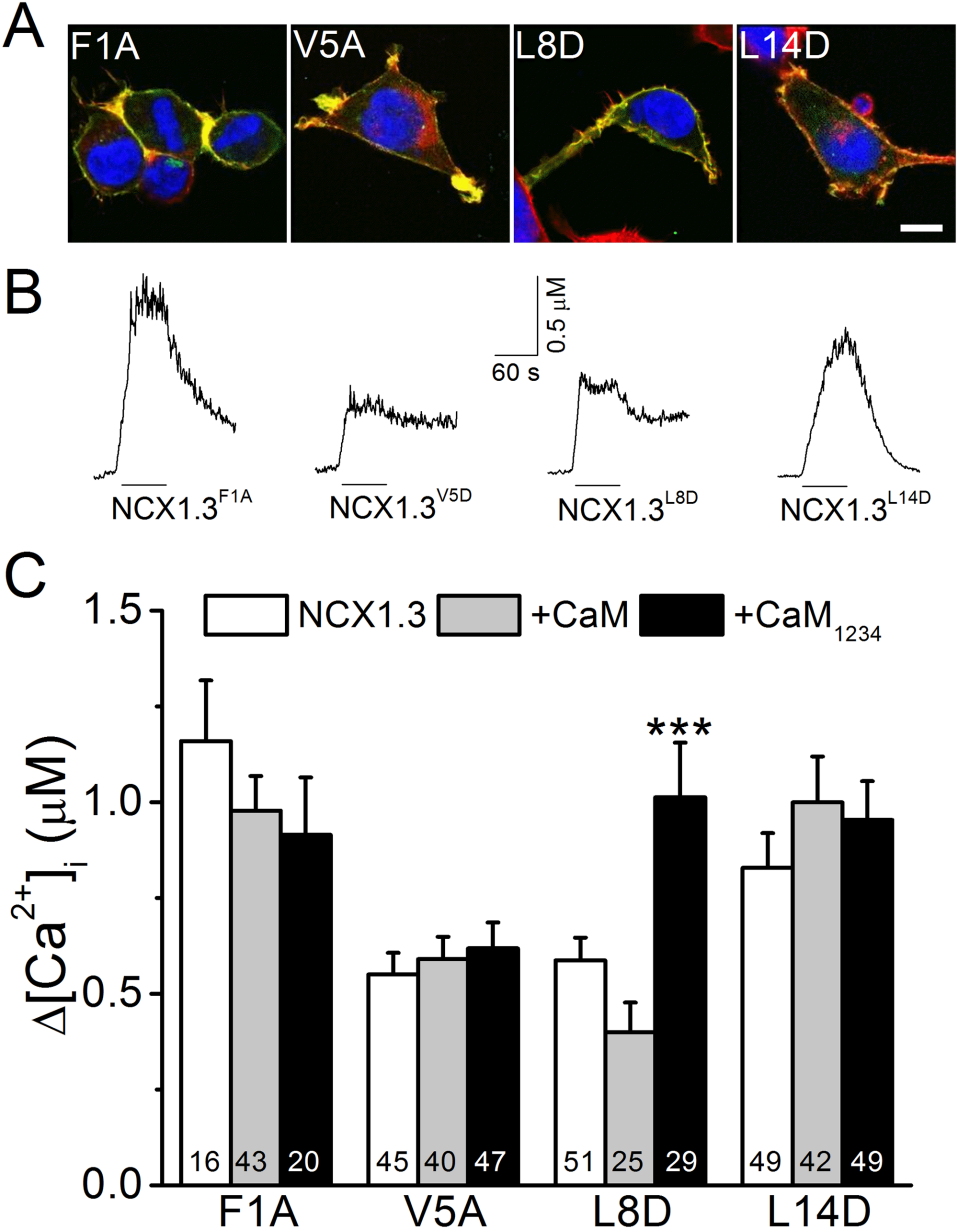
Mutations in the conserved a.a. residues of the NCX1.3 CaMS affect exchange activity. The conserved 1^st^, 5^th^, 8^th^, and 14^th^ a.a. residues of the CaMS of NCX1.3 was independently mutated from F, V, L, and L, to A (F1A), A (V5A), D (L8D), and D (L14D), respectively. We measured the reverse-mode exchange activity of HEK293T cells expressing constructs with or without co-expression of CaM or CaM_1234_. A. Merged images of cells expressing various NCX1.1 mutants stained with phalloidin (Red), DAPI (Blue), and an antibody against the V5 epitope tag (Green) to visualize actin filaments, nuclear DNA, and the exchanger, respectively. Scale bar: 10 μm. B. The [Ca^2+^]_i_ responses of single HEK293T cells expressing various constructs. The black lines under each trace indicate the period of NMG perfusion. C. Average [Ca^2+^]_i_ elevations in cells expressing various NCX1.3 mutants with or without co-expression of CaM or CaM_1234_. The digits in each column indicate the number of cells in each group. Data shown are the mean ± SEM pooled from three different batches of cells and analyzed by a one-way ANOVA with Fisher's post hoc test (***: *p* < 0.001 when compared with the corresponding group expressing only the NCX1.3 mutant).

The 4 conserved a.a. residues of the CaMS in NCX1.3 were individually mutated as F1A, V5A, L8D, and L14D to characterize the roles of these a.a. residues in modulating NCX1.3 activity (Fig 7). Immunostaining revealed that these NCX1.3 mutants were present at the cell boundary and that they overlapped with F-actin staining. The line intensity profiles also suggested a plasma membrane distribution for these mutants (S4 Fig). These mutants all displayed rNCX activity upon NMG perfusion, but NCX1.3^V5A^ and NCX1.3^L8D^ had lower [Ca^2+^]_i_ elevations than those of NCX1.3^F1A^ and NCX1.3^L14D^. The average [Ca^2+^]_i_ changes in cells expressing NCX1.3^F1A^ and NCX1.3^L14D^ were 1160 ± 158 (n = 16) and 829 ± 90 (n = 49) nM, respectively, similar to that of NCX1.3 (902 ± 100 nM, Fig 4C). In contrast, the NCX1.3^V5A^ and NCX1.3^L8D^ had a significantly smaller [Ca^2+^]_i_ changes of 550 ± 56 (n = 45, *p* < 0,01) and 587 ± 59 (n = 51, *p* < 0,01) nM, respectively, compared with the wild type (Fig 4C). CaM and CaM_1234_ co-expression did not affect the exchange activity of these mutants, except for NCX1.3^L8D^, whose activity was slightly, but not significantly attenuated to 400 ± 78 nM (n = 25, *p* = 0.07) by CaM and significantly increased to 1013 ± 142 nM (n = 29, *p* < 0.001) by CaM_1234_. These results demonstrate that these conserved a.a. residues in the CaMS have differential effects on CaM-mediated regulation.

## Discussion

CaM is a multi-functional Ca^2+^-sensing protein that regulates a variety of physiological activities by interacting with different proteins [26,27,28]. Although CaM interacts with several Ca^2+^-related ion channels and transporters to regulate Ca^2+^ homeostasis, it is not clear whether CaM interacts with NCX or NCKX, which are important for exporting Ca^2+^ out of cells. This report demonstrates that the predicted 1-5-8-14 CaM binding motif in NCX1 splice variants is important for Ca^2+^ transport and membrane localization; in addition, pull-down assays verify the interaction between CaM and NCX1 splice variants. Therefore, CaM may interact with the intracellular loop of NCX1 splice variants to regulate exchange activity.

Deleting the CaMS from NCX1 splice variants attenuates the exchange activity and interferes with the membrane targeting of these NCX1 splice variants. When co-expressed with the NCX1 splice variants without CaMS, the membrane targeting of GFP-GPI is also blocked, suggesting that the overexpression of NCX1 splice variants that lack the CaMS may block the secretory pathway and prevent other membrane proteins from reaching the plasma membrane. It is likely that the decrease in exchange activity is associated with the attenuated membrane expression level. In contrast, immunostaining of NCX1.1 and NCX1.3 mutants with point mutations in the CaMS show that these mutants localize mostly to the plasma membrane. Therefore, the whole CaMS is important for the correct targeting of NCX1 splice variants to the plasma membrane.

The binding of CaM to the 1-5-8-14 motif of the Ca^2+^-ATPase releases the auto-inhibitory domain and enhances transport activity [17]. For IP_3_ receptors, an unidentified Ca^2+^-binding motif is thought to compete with CaM in binding to the endogenous 1-5-8-14 motif for channel activation [29]. Recently, a structural study characterizing the interaction between CaM and the 1-13-16 motif of the inositol 1,4,5-trisphosphate 3-kinase suggests that the recruitment of CaM to the kinase is determined by multiple interaction domains rather than a particular a.a residue [30]. In the present report, GST-CaM pull-down assays confirm that CaM undergoes a Ca^2+^-dependent interaction with the intracellular loop of NCX1 splice variants through the CaMS. In addition, the XIP at the N-terminus of the NCX1.1 intracellular loop may interact with a region at a.a. 566–679 of NCX1.1, which covers the CBD2 and the alternative splicing region [31,32]. It is possible that NCX1 splice variants have an endogenous 1-5-8-14 binding motif that competes with CaM to regulate the exchange activities, though this needs to be further verified. Since partial intracellular loop is used for the pulldown assay, we could not exclude the possibility that, in the absence of CaMS, CaM might bind to the NCX1 splice variants with a weak or transient association through the interactions with other motifs. The pulldown assay could not verify this weak interaction but the activity assays using full-length constructs could. Therefore, we postulate that XIP, CBDs, CaM, and CaMS may interact with each other to form a stable loop complex and support exchange activity [13,33].

The Ca^2+^-binding affinities of CBD1 and CBD2 are approximately 0.2 and 5 μM, respectively [13]; for CaM, the affinity is approximately 1 μM [34]. In addition, exon A in the CBD2 domain has the ability to bind Ca^2+^ and stabilize the loop complex, whereas exon B lacks Ca^2+^ binding ability and may destabilize the complex [35,36]. Therefore, the alternative splicing exons in CBD2 regulate the Ca^2+^ binding affinities of CBD1/2 to meet the physiological requirements in different tissues under different [Ca^2+^]_i_ levels [36]. The intracellular loops of NCX1.1 and NCX1.4, which contain exon A, may have already obtained a stable loop complex, with CaM binding playing a minor role in regulating exchange activity, because overexpressed CaM and CaM_1234_ had no effect on the exchange activity of these two constructs. In contrast, due to the destabilizing effect of exon B, endogenous CaM binding is important for NCX1.3 to obtain a stable loop complex; the overexpressed CaM_1234_ may interfere with this interaction and decrease exchange activity. Therefore, how CaM interacts cooperatively with CBD1 and CBD2 of different alternative splicing exons to modulate exchange activity under different [Ca^2+^]_i_ levels requires further investigation.

Our results reveal that each conserved a.a. in the CaMS contributes differentially to the exchange activities of NCX1.1 and NCX1.3. For NCX1.1, mutating the first 2 conserved a.a. residues in the CaMS of NCX1.1 may directly destabilize the loop complex and down-regulate exchange activity. Although mutations in the 3^rd^ and 4^th^ conserved a.a. residues did not affect exchange activity, these mutations may weaken the loop complex, and the binding of overexpressed CaM, but not CaM_1234_, destabilizes the loop complex to reduce exchange activity. In summary, the first 2 conserved a.a. residues of CaMS in NCX1.1 may play a dominant role in stabilizing the structure to maintain exchange activity; the 3^rd^ and 4^th^ conserved a.a. residues may be involved in the access of CaM to this loop complex.

For the NCX1.3, which contains exon B, CaM binding may be required to stabilize the loop complex and support exchange activity. Mutating the 1^st^ and 4^th^ conserved a.a. residues in the CaMS of NCX1.3 does not affect exchange activity, suggesting that these mutations may not affect CaM binding. In contrast, the 2^nd^ and 3^rd^ conserved a.a. residues are the main residues involved in determining structural stability and CaM binding; therefore, mutations in these two residues decreased exchange activity. Since CaM_1234_ does not bind to the cytosolic loop, it is not clear how CaM_1234_ overexpression rescues the exchange activity of NCX1.3^L8D^. It is possible that CaM_1234_ transiently interacts with the loop complex and interferes with the interaction between endogenous CaM and the loop complex. How the CaMS interacts with other domains to modulate CaM binding will require further studies on the structure.

In intact cardiomyocytes, allosteric regulation of NCX1 activity by cytosolic Ca^2+^ is highly cooperative, related to the binding of Ca^2+^ to the CBD motifs, but exhibits slow activation kinetics, with a 20–50 msec lag-phase to reach maximal exchange activity [37,38]. CaM has a Ca^2+^ affinity of approximately 1 μM, which is in between the affinities of CBD1 and CBD2. Hence, the cooperation among CaM, CBD1, and CBD2 in response to various [Ca^2+^]_i_ levels during excitation may help tune NCX1 activity. The lag in reaching maximal exchange activity may be due to the time required to form a stable loop complex among these Ca^2+^-binding motifs. Therefore, CaM is involved in regulating not only structural stability but also temporal control. In addition, NCX isoforms are extremely sensitive to mild cytosolic acidification; a pH decrease from 7.2 to 6.9 results in nearly 90% inactivation of NCX, called "proton block" [37,39]. Given that the protonation of CaM decreases its Ca^2+^ affinity, pH decreases from 7.2 to 6.96 result in a decrease in p*K*_*Ca*_ from 6.02 ± 0.04 to 5.51 ± 0.04 [34]. Therefore, the sensitivity of CaM to pH in terms of its Ca^2+^ binding may be related to the mechanism behind the proton block of NCX activity. These correlations suggest a possible interaction between CaM and NCX1 splicing variants in regulating exchange activity under physiological conditions.

The mutually exclusive exons A and B are important for exchange activity. Under high levels of [Ca^2+^]_i_, exon A, but not B, releases the Na^+^-dependent inactivation of exchange activity [14]. Some pharmacological experiments also show differential sensitivities of NCX1.1 and NCX1.3 splice variants to fatty acids in regulating Ca^2+^ homeostasis [40]. Without exon A, exchange activity of NCX1D is greatly reduced. Because the alternative splicing region is part of CBD2, different exon combinations may affect the Ca^2+^ affinity of CBD2, which regulates the stability of the loop complex and exchange activity. Therefore, these domains in the intracellular loop of NCX1 splice variants may form a complex that depends on the Ca^2+^ concentration and exon expressed.

The activation of ionotropic receptors in excitable cells allows the influx of Na^+^ ions and depolarizes the membrane, which favors rNCX activity to transport Ca^2+^ into the cytosol. This Ca^2+^ influx triggers the Ca^2+^-induced Ca^2+^ release pathway to maintain high [Ca^2+^]_i_ and support various physiological functions [41,42]. Therefore, the involvement of CaM in supporting rNCX activity may facilitate the fine-tuning of rNCX activity under different [Ca^2+^]_i_ levels to regulate Ca^2+^ homeostasis. However, it is not clear whether CaM plays the same role in modulating forward exchange activity, and this question needs to be studied further.

NCX isoforms expressed in different tissues may contribute differentially in modulating Ca^2+^ homeostasis [12,43,44]. In addition to the dynamic response of the CBD1/2 structure to [Ca^2+^]_i_ changes [36], our results demonstrate that the interaction among the alternative splicing region, CaM, and CaMS modulates NCX1 activity. Hence, different levels of [Ca^2+^]_i_ elevations will regulate NCX activity accordingly to modulate Ca^2+^ homeostasis. NCX isoforms are involved in heart disease and brain damage, and this interaction may provide new insights for the selective pharmacological targeting of NCX isoforms.

## Supporting Information

S1 Fig Stained intracellular loop after Triton X-100 permeabilization. HEK293T cells expressing NCX1.1CL or NCX1.3CL were fixed and permeabilized by triton-X100; then the antibody against V5 epitope tagged at the C-terminals of these loop proteins was applied to visualize the expressed proteins. Scale bar: 10 μm.
(TIF)

S2 Fig CaMS deletion interferes the membrane localization of NCX1 splice variants. The NCX1 splice variants NCX1.1 (A) and NCX1.3 (B) with or without CaMS, NCX1.1ΔCaMS (C) and NCX1.3ΔCaMS (D), were coexpressed with GFP-GPI (Green) in HEK293T cells. After permeabilization, the NCX variants and mutants were immunostained (Red), and images were taken via confocal microscopy. The arrows indicate the region used to plot the line intensity profile. Scale bar: 10 μm.
(TIF)

S3 Fig Distribution of NCX1 mutants at the cell membrane. The conserved 1^st^, 5^th^, 8^th^, and 14^th^ a.a. residues at the CaMS of the A. NCX1.1 and B. NCX1.3 were independently mutated from F, V, L, and L, to A (F1A), A (V5A), D (L8D), and D (L14D), respectively. The images (upper row) shows the merged fluorescence of cells expressing various mutants stained with phalloidin (Red), DAPI (Blue), and antibody against V5 epitope (Green). The lower rows plotted the intensity profiles indicate the red and green fluorescence intensity along the arrows in the corresponding merged images. Scale bar: 10 μm.
(TIF)
